# Survival benefit evaluation of radiotherapy in esophageal cancer patients aged 80 and older

**DOI:** 10.18632/oncotarget.22884

**Published:** 2017-12-04

**Authors:** Shan Huang, Shuyu Zheng, Tuotuo Gong, Hongbing Ma, Yue Ke, Songchuan Zhao, Wenyu Wang, Lijun Jia, Xiaozhi Zhang

**Affiliations:** ^1^ Department of Radiotherapy, Second Affiliated Hospital, Xi’an Jiaotong University, Xi'an, China; ^2^ Department of Radiotherapy, First Affiliated Hospital, Xi’an Jiaotong University, Xi'an, China; ^3^ Department of Spine Surgery, Honghui Hospital, Health Science Center, Xi’an Jiaotong University, Xi'an, China; ^4^ Institute for Molecular Medicine Finland (FIMM), University of Helsinki, Helsinki, Finland; ^5^ Department of Oncology, Second Affiliated Hospital, Xi’an Jiaotong University, Xi'an, China

**Keywords:** esophageal cancer, aged 80 and older, radiotherapy, survival, stage

## Abstract

**Purpose:**

To evaluate the survival benefit of radiotherapy (RT) in esophageal cancer (EC) patients aged ≥ 80.

**Materials and Methods:**

Records for all EC patients aged ≥ 65 years were extracted from the Surveillance, Epidemiology, and End Results database. Chi-square test compared the characteristic and treatment between patients aged ≥ 80 with those aged 65–79. Focusing on patients aged ≥ 80, we employed multivariable logistic regression to identify the association between selection of RT and patients’ characteristics. Survival curve was employed to visualize the survival rate and multivariable Cox proportional hazard model was established to quantify the effect of RT on overall survival (OS) and cancer special survival (CSS).

**Results:**

Patients aged ≥ 80 were more likely to be white male and have localized EC (all *P* < 0.001). Selection of RT in patients aged ≥ 80 were associated with cancer histology (*P* < 0.001), grade (*P =* 0.024) and stage (*P* < 0.001). RT significantly improved the OS (hazard ratio(HR) = 0.717) and CSS (HR = 0.722) (all *P* < 0.001). Further stratified analysis found the improvement were only significant in the localized (OS HR = 0.662; CSS HR=0.652) and regional stage patients (OS HR = 0.571; CSS HR = 0.581) (all *P* < 0.001).

**Conclusions:**

Our study suggested EC patients aged ≥ 80 benefit from RT only if the cancer is in localized/regional stage.

## INTRODUCTION

Esophageal cancer (EC) is the one of the most common and fatal malignant tumor worldwide [1]. It is estimated over 15,000 Americans will die from EC in 2017 [2]. EC patients have a median age of 67 years at diagnosis [3, 4]. Aging of population leads to elder EC patients these years. This elder group need special recommendations on treatment and medical care [5].

Studying the prognosis factors and treatment benefit of EC patients aged ≥ 80 may help patients and clinicians make decisions that are more appropriate. For example, radiotherapy (RT) constitutes one of the most important treatment for EC patients. However, the related adverse reactions of RT, such as pneumonia, cardiac toxicity, electrolyte imbalance, bone marrow suppression, pulmonary and even systemic infection, often staggers the choice of using it [6]. A clear evaluation about survival benefit of RT provides trustworthy reference for both patients and clinicians. Prior studies have evaluated the survival benefit of RT in EC patients aged 65–79 [7–9], yet whether and to what extent RT brings survival benefits to EC patients aged ≥ 80 remains to be confirmed [10].

The Surveillance, Epidemiology, and End Results (SEER) Program of the National Cancer Institute (NCI) provides an authoritative source of information about cancer incidence and survival in the United States. SEER currently collects and publishes cancer incidence and survival data from population-based cancer registries covering approximately 28% of the U.S. population. The SEER program is the only comprehensive source providing information about stage of the cancer at the time of diagnosis and patient survival data [11].

In the present study, we conducted a retrospective population-based study for EC patients aged ≥ 80 using SEER database. We explored clinical characteristics, prognosis factors and efficacy of RT for EC patients aged ≥ 80. This study represents the first large sample size based descriptive analysis of EC patients aged ≥ 80.

## RESULTS

### Characteristics difference between EC patients aged 65–79 with aged ≥ 80

A total of 12,407 EC patients were enrolled in the present study, including 9,946 patients aged 65–79, and 2,461 patients aged ≥ 80 (19.8%). Characteristics of each age category were displayed in Supplementary Table 1. Both the number and the proportion of EC patients aged ≥ 80 had increased steadily these years. Compared with patients aged 65–79, EC patients aged ≥ 80 were more likely to be white male and have localized EC (all *P* < 0.001). Surgery treatment rate declined dramatically with age. Among EC patients aged ≥ 80 years, up to 83.9% patients had not been treated with surgery, while over two thirds of patient ultimately received RT. The median survival time (MST) of patients aged over 80 significantly shorter than those aged 65–79 (10 vs. 12 months, *P* < 0.001). Same trend was also shown in 1, 3, 5-year overall survival rate.

We investigated the survival rate, clinicopathologic characteristics including stage distribution and treatment status of EC patients aged ≥ 80 years at the different periods of time. Among EC patients aged ≥ 80 years diagnosed between 2004–2013, only 14.4% patients had been treated with surgery, while 64.0% patient ultimately received RT. The RT served as the main treatment for the patients all over the period. The MST and 1, 3, 5-year overall survival rate increased over the years. Detailed characteristics were presented in Supplementary Table 2.

### Characteristic associated with RT selection in EC patients aged ≥ 80

We further investigated possible characteristics associated with RT in patients aged ≥ 80. We removed the patients treated by surgery to exclude its interference. A total of 2,066 EC patients aged ≥ 80 were eligible for further analysis by multivariable logistic regression analysis and models were adjusted for potential confounders listed in Table 1. Our results showed the proportion of patients treated by RT decreased significantly across 40 years period (1994–2003, odds ratio (OR) = 0.582; 2004–2013, OR = 0.450). Selection of RT were associated with histology (*P* < 0.001), grade (*P* = 0.024) and stage (*P* < 0.001). Patients with regional stage cancer were more likely to receive RT as compared to patients with localized (81.2% vs. 72.2%, OR = 1.880), but a reverse trend was seen in distant stage (OR = 0.757).

**Table 1:** Association between selection of RT and characteristics of EC patients aged ≥ 80

Characteristic	No. (%) of patients						
	RT (*n =* 1514)	NRT (*n =* 552)	*P*		OR^*^	95% CI	
**Year of diagnosis**			< 0.001				
1973–1993	204 (84.0)	39 (16.0)	Reference		1		
1994–2003	463 (75.9)	147 (24.1)	0.008		0.582	0.391–0.868	
2004–2013	847 (69.8)	366 (30.2)	< 0.001		0.450	0.308–0.657	
**Race**			0.372				
White	1333 (73.2)	488 (26.8)	Reference		1		
Black	87 (71.9)	34 (28.1)	0.160		0.733	0.475–1.131	
Other	94 (75.8)	30 (24.2)	0.817		0.949	0.608–1.482	
**Sex**			0.248				
Female	524 (73.6)	188 (26.4)			1		
Male	990 (73.1)	364 (26.9)			1.137	0.915–1.413	
**Histology**			< 0.001				
SC	743 (78.3)	206 (21.7)			1		
AD	771 (69.0)	346 (31.0)			0.620	0.496–0.774	
**Grade**			0.024				
Well	78 (70.3)	33 (29.7)	Reference		1		
Moderately	655 (75.7)	210 (24.3)	0.074		1.508	0.962–2.363	
Poorly or Un	781 (71.7)	309 (28.3)	0.521		1.156	0.742–1.801	
**Stage**			< 0.001				
Localized	606 (72.2)	233 (27.8)	Reference		1		
Regional	579 (81.2)	134 (18.8)	< 0.001		1.880	1.467–2.409	
Distant	329 (64.0)	185 (36.0)	0.026		0.757	0.591–0.968	

Abbreviation: RT, radiotherapy; EC, esophageal cancer; SC, squamous cell carcinoma; AD, adenocarcinoma; OR, odds ratio; CI, confidence interval.

^*^ORs were derived from multivariable logistic regression analysis, and models were adjusted for all confounding factors listed in the table.

### Survival benefit evaluation of RT in EC patients aged ≥ 80

**Figure 1:**
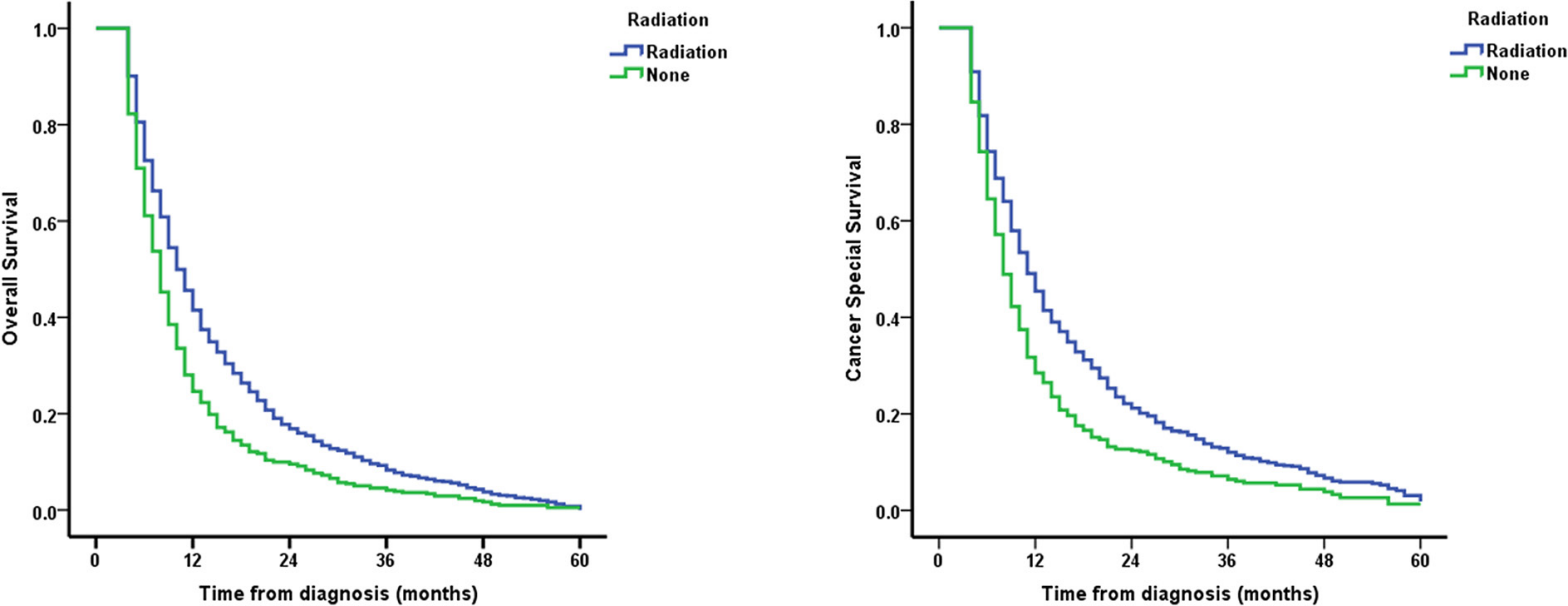
Survival curves of EC patients aged ≥ 80

As shown in Figure 1, comparing to NRT group, patients receiving RT presented longer overall survival (OS) and cancer special survival (CSS). Adjusted for potential confounders, we conducted multivariate Cox regression analysis. The variables associated with survival of EC patients aged ≥ 80, including year of diagnosis, race, sex, and histology grade were controlled. We found that stage and RT served as main prognostic factors for EC patients aged ≥ 80 (Table 2). Patients with regional stage (hazard ratio (HR) = 1.215, 95% confidence interval (CI), 1.091–1.352) and distant stage (HR = 1.506, 95% CI, 1.336–1.697) tended to have worse OS compared to patients with localized stage. A similar trend was shown in CSS based analysis (all *P* < 0.001). No significant association was found for histology and grade. Patients who received RT showed better prognosis compared to patients without RT (all *P* < 0.001 (HR = 0.717, 95% CI, 0.646–0.797 for OS and HR = 0.722, 95% CI, 0.646–0.807 for CSS, respectively).

**Table 2:** Cox proportional hazards analysis of variables associated with survival of EC patients aged ≥ 80

Characteristic	OS			CSS		
	*P*	HR^*^	95% CI	*P*	HR^*^	95% CI
**Year of diagnosis**						
1973–1993	Reference	1		Reference	1	
1994–2003	0.244	0.913	0.784–1.064	0.044	0.848	0.722–0.995
2004–2013	0.001	0.783	0.675–0.907	< 0.001	0.734	0.629–0.857
**Race**						
White	Reference	1		Reference	1	
Black	0.214	1.133	0.931–1.378	0.197	1.149	0.930–1.419
Other	0.830	1.021	0.844–1.236	0.368	1.096	0.898–1.339
**Sex**						
Female	Reference	1		Reference	1	
Male	0.506	0.967	0.877–1.067	0.304	0.947	0.853–1.051
**Histology**						
SC	Reference	1		Reference	1	
AD	0.483	1.036	0.938–1.144	0.070	1.103	0.992–1.227
**Grade**						
Well	Reference	1		Reference	1	
Moderately	0.504	0.932	0.756–1.147	0.394	0.908	0.726–1.134
Poorly or Un	0.555	1.064	0.866–1.307	0.476	1.083	0.869–1.349
**Stage**						
Localized	Reference	1		Reference	1	
Regional	< 0.001	1.215	1.091–1.352	< 0.001	1.239	1.105–1.391
Distant	< 0.001	1.506	1.336–1.697	< 0.001	1.597	1.406–1.813
**RT**						
None	Reference	1		Reference	1	
RT	< 0.001	0.717	0.646–0.797	< 0.001	0.722	0.646–0.807

Abbreviations: EC, esophageal cancer; OS, overall survival; CSS, cancer special survival; SC, squamous cell carcinoma; AD, adenocarcinoma; RT, radiotherapy; HR, hazard ratio; CI, confidence interval.

^*^HRs were derived from multivariable Cox proportional hazards regression analysis, and models were adjusted for all confounding factors listed in the table.

To further specify the benefit in patient subgroup, stratified Cox regression analysis was conducted to evaluate the impact of RT on OS and CSS by stage, adjusting for year of diagnosis. As presented in Table 3, RT significantly improved localized patients both in OS (HR = 0.662, 95% CI, 0.563–0.779) and CSS (HR = 0.652, 95% CI, 0.548–0.776). Consistent results were also observed in regional patients. For distant patients, we did not observe significance difference in both OS (HR = 0.941, *P* = 0.527) and CSS (HR = 0.966, *P* = 0.736).

**Table 3:** Impact of RT on overall survival and cancer special survival by stage of EC patients aged ≥ 80

Stage	OS		CSS		
	HR (95% CI)	*P*	HR (95% CI)		*P*
**Localized**					
RT vs. None	0.662 (0.563–0.779)	< 0.001	0.652 (0.548–0.776)	< 0.001	
**Regional**					
RT vs. None	0.571 (0.468–0.698)	< 0.001	0.581 (0.470–0.718)	< 0.001	
**Distant**					
RT vs. None	0.941 (0.778–1.137)	0.527	0.966 (0.792–1.179)	0.736	

Abbreviations: RT, radiotherapy; EC, esophageal cancer; OS, overall survival; CSS, cancer special survival; HR, hazard ratio; CI, confidence interval.

HR and *P* values were determined from multivariable Cox proportional hazards regression analysis adjusting for year of diagnosis.

**Figure 2:**
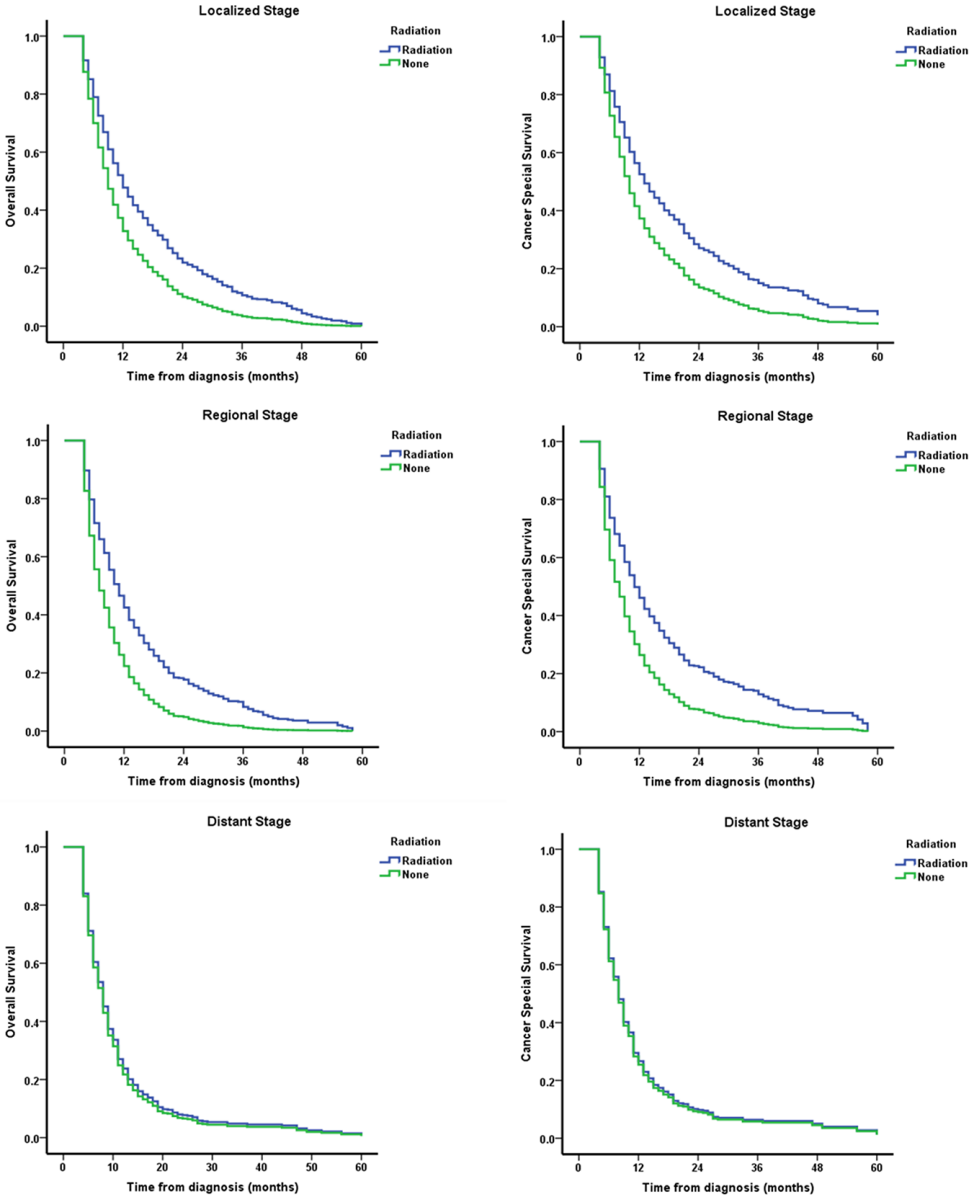
Survival curves of EC patients aged ≥ 80 stratified by different disease stages and adjusted for year of diagnosis

Finally, we delineated the effect of RT on survival in EC patients aged ≥ 80 using stratified survival curves. As shown in Figure 2, RT significantly improved the patients’ OS and CSS in localized and regional cases, but not in cases with distant stage which validated this finding.

## DISCUSSION

RT is the most important therapy for EC patients aged ≥ 80. However, the survival benefit of RT for this group has yet not been well evaluated. Using large national cohort data from SEER database, we compared the clinical and pathological characteristics of patients aged ≥ 80 to those aged 65–79. In addition, we also analyzed the prognostic factor as well as survival benefit of RT in EC patients aged ≥ 80. To the best of our knowledge, our study constitutes the first large database based observational study focusing on RT benefit of EC patients aged ≥ 80.

Comparison of the clinical and pathological characteristics revealed several different characteristics for patients aged ≥ 80. As anticipated, the number and proportion of EC patients aged ≥ 80 increased significant across period. An interesting finding was, although most of EC patients aged ≥ 80 were still male, the female ratio had increased comparing with that of 65–79. This result was consistent with the previous report [12] that the female patients of EC are older than male patients [11].

RT and surgery are two main treatments strategies for EC, especially localized EC [12]. Although there were more localized patients in EC patients aged ≥ 80 in the cohort, we found a significant decrease of surgery rate and RT overweighed surgery as the main treatment for this group of patients. Our findings confirmed that the importance of RT in EC patients aged ≥ 80. We also evaluated the survival benefit of RT in this special group and survival analysis showed that RT was prognostic factor of OS and CSS. In multivariate analysis, RT increased both OS and CSS, and benefit of CSS was slightly higher than that of OS. Previous studies reported elderly patients with EC including those aged ≥ 80 benefit from RT [13–15]. Our findings confirmed the therapeutic benefit for EC patients aged ≥ 80 in a much larger sample size.

Our survival analysis demonstrated the importance of stage information when choosing RT for EC patients aged ≥ 80. RT served as the standard treatment plan for localized EC [16, 17]. Previous retrospective study reported that surgery plus preoperative RT improved the OS of patients with metastatic thoracic EC [18]. Our results showed, for EC patients aged ≥ 80, only those with localized/regional EC will benefit from RT. No survival benefit has been observed for patients with distant stage EC after controlling for confounding factor. Noteworthy, over 60% of patients with distant stage EC received RT, and still considerable proportion of patients with localized/regional EC did not receive RT. Our results suggested the importance of cancer stage information in therapy selection for EC patients aged ≥ 80. Further studies are warranted to confirm and interpret this finding.

Only a very few number of EC patients aged ≥ 80 received surgery. To focus on evaluating the benefit of RT, we ruled out this group of patients. It should be noted that, due to the lack of medical information in the SEER database, we were unable to obtain details of chemotherapy. Potential chemotherapy may influence survival, which lead to biased results. However, patients aged ≥ 80 are mostly unlikely to tolerate chemotherapy [19, 20]. We have also excluded patients followed up less than 4 months to minimize the effect of chemotherapy in our survival analysis.

In conclusion, our study provided creditable evidence for characteristics and outcome for EC patients aged ≥ 80 based on large, population-based registry. Our study suggested that EC patients aged ≥ 80 benefit from RT only if the cancer is in localized/regional stage.

## MATERIALS AND METHODS

### Data source

Public SEER data from 1973 to 2013 [“Incidence - SEER 18 Regs Research Data + Hurricane Katrina Impacted Louisiana Cases, Nov 2015 Sub (1973–2013 varying)”] was extracted for the study (reference number: 12420-Nov2015). The latest release of SEER*Stat Software (Version 8.3.2) was used to clean the raw SEER data [3]. No human sample or personal identifying information was involved in this study.

### Patient selection criteria

Patients diagnosed with primary EC and aged ≥ 65 years were eligible for analysis. EC cases were confirmed using the World Health Organization International Classification of Disease for Oncology 3rd Edition (ICD-O-3, site rode: C15.0-C15.5 and C15.8-C15.9). Disease stages were defined by SEER historic stage criteria, including localized (invasive but confined to organ of origin), regional (extension beyond organ of origin but no distant metastasis), or distant (distant metastasis) [21]. Histologic types were identified using ICD-O-3 histology code.

Inclusion criteria contained: (1) diagnostic confirmation by positive histology; (2) active follow-up; (3) only one malignant primary indicator. Individuals were excluded with: (1) unknown age, sex, grade, race, SEER historic stage; (2) surgery status of “Recommended, unknown if performed”, “unknown”, “death certificate” or “autopsy only”; (3) RT of “Recommended, unknown if performed” or “Unknown”.

### Overall survival and cancer special survival

Survival time was evaluated using evaluated OS and CSS. Survival data was extracted at 1-month intervals for a minimum follow-up of 4 months and a maximal follow-up of 60 months to minimize the effect of chemotherapy and exclude patients not survive long enough to receive cancer-directed therapy. OS was determined from the SEER records of survival time (total No. of months) and vital status. CSS was determined from the SEER cause-specific death classification variable and was evaluated from the date of diagnosis to the date of EC specific death.

### Statistical analysis

**Figure 3:**
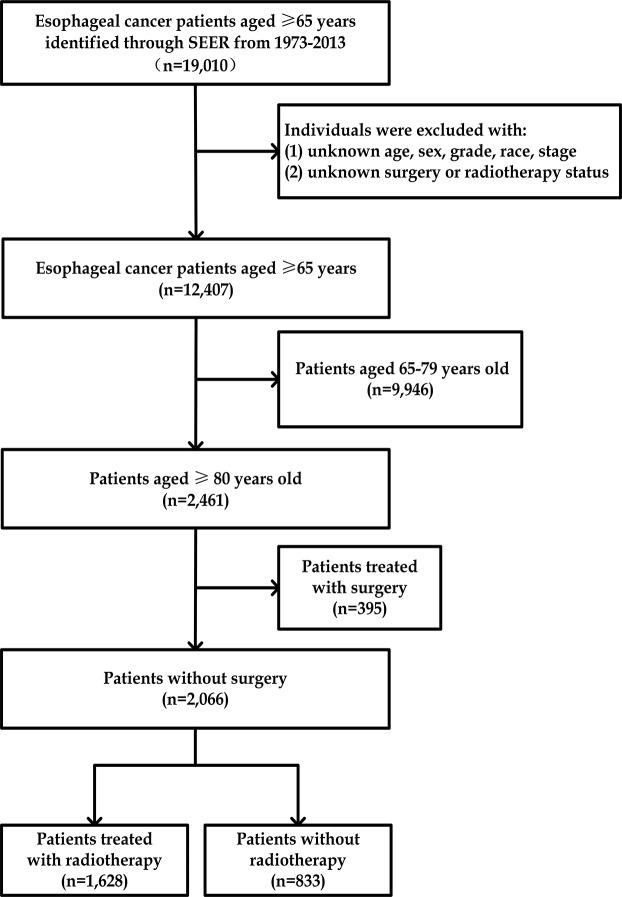
Study workflow

Enrolled patients were first divided into 2 subgroups by age: 65–79 and over 80. Patient’s characteristics and treatment were compared by Chi-square test. Subsequently, focusing on survival evaluation of RT, patients aged between 65–79 and patients who received surgery were ruled out from further analysis (Figure 3). Multivariable logistic regression was employed to investigate the potential influencing factors on patients’ selection upon RT. Multivariable Cox proportional hazard model was established to quantify the effect of RT on OS and CSS adjusting for year of diagnosis, race, sex, histology grade and stage. Survival curve was employed to visualize the survival condition of RT and NRT group. Finally, staged stratified survival was delineated by survival curve and Cox regression adjusted for the same variables described above. Significance was identified according to predefined threshold 0.05. All statistical analyses were implemented using the statistical analysis software SPSS 22.0 for Windows (SPSS, Inc., Chicago, IL, USA).

## SUPPLEMENTARY MATERIALS TABLES


