# Supplementary material for: Survival benefit evaluation of radiotherapy in esophageal cancer patients aged 80 and older
Source: Oncotarget. 2017 Dec 4;8(67):112094–102. doi: 10.18632/oncotarget.22884 (PMC5762382; doi:10.18632/oncotarget.22884)
Supplement: Supplementary file 1 [file oncotarget-08-112094-s001.pdf]

# Survival benefit evaluation of radiotherapy in esophageal cancer patients aged 80 and older

## SUPPLEMENTARY MATERIALS

**Supplementary Table 1: Characteristics and treatment of EC patients aged ≥ 80**

| Characteristic | Age at diagnosis 65-79 (*n* = 9946) | ≥ 80 (*n* = 2461) | Total (*n* = 12407) | $\chi^2$ | *P*[*] |
|---|---|---|---|---|---|
| **MST (months)** | 12.00 | 10.00 | | 52.415 | < 0.001 |
| **Overall survival (%)** | | | | | |
| 1-year | 46.9 | 40.1 | | | |
| 3-year | 12.3 | 8.6 | | | |
| 5-year | 0.5 | 0.1 | | | |
| **Year of diagnosis** | | | | 50.198 | < 0.001 |
| 1973-1993 | 1817 (18.3) | 310 (12.6) | 2127 (17.1) | | |
| 1994-2003 | 2992 (30.1) | 734 (29.8) | 3726 (30.0) | | |
| 2004-2013 | 5137 (51.6) | 1417 (57.6) | 6554 (52.8) | | |
| **Race** | | | | 54.127 | < 0.001 |
| White | 8321 (83.7) | 2181 (88.6) | 10502 (84.6) | | |
| Black | 1004 (10.1) | 132 (5.4) | 1136 (9.2) | | |
| Other | 621 (6.2) | 148 (6.0) | 769 (6.2) | | |
| **Sex** | | | | 136.362 | < 0.001 |
| Female | 2245 (22.6) | 835 (33.9) | 3080 (24.8) | | |
| Male | 7701 (77.4) | 1626 (66.1) | 9327 (75.2) | | |
| **Histology** | | | | 4.634 | 0.099 |
| SC | 4608 (46.3) | 1081 (43.9) | 5689 (45.9) | | |
| AD | 5329 (53.6) | 1378 (56.0) | 6707 (54.1) | | |
| Other | 9 (0.1) | 2 (0.1) | 11 (0.1) | | |
| **Grade** | | | | 3.841 | 0.147 |
| Well | 588 (5.9) | 146 (5.9) | 734 (5.9) | | |
| Moderately | 3937 (39.6) | 1026 (41.7) | 4963 (40.0) | | |
| Poorly or Un | 5421 (54.5) | 1289 (52.4) | 6710 (54.1) | | |
| **Stage** | | | | 176.024 | < 0.001 |
| Localized | 2754 (27.7) | 1002 (40.7) | 3756 (30.3) | | |
| Regional | 4001 (40.2) | 904 (36.7) | 4905 (39.5) | | |
| Distant | 3191 (32.1) | 555 (22.6) | 3746 (30.2) | | |
| **Surgery** | | | | 300.883 | < 0.001 |
| Surgery | 3384 (34.0) | 395 (16.1) | 3779 (30.5) | | |
| None | 6562 (66.0) | 2066 (83.9) | 8628 (69.5) | | |
| **RT** | | | | 3.001 | 0.083 |
| RT | 6761 (68.0) | 1628 (66.2) | 8389 (67.6) | | |
| None | 3185 (32.0) | 833 (33.8) | 4018 (32.4) | | |

Abbreviations: EC, esophageal cancer; MST, median survival time; RT, radiotherapy.
MST and 1, 3, 5-year survival rate were determined by Kaplan-Meier Analysis.
[*]MST: log-rank test; others: Chi-square test.

**Supplementary Table 2: Characteristics and treatment of EC patients aged ≥80 at the different periods of time**

| | Year of diagnosis | | | | | |
|---|---|---|---|---|---|---|
| **Characteristic** | **1973–1993 (*n* = 310)** | **1994–2003 (*n* = 734)** | **2004–2013 (*n* = 1417)** | **Total (*n* = 2461)** | $\chi^2$ | $P^*$ |
| **MST (months)** | 9.00 | 10.00 | 11.00 | | 15.575* | < 0.001* |
| **Overall survival (%)** | | | | | | |
| 1-year | 36.1 | 37.5 | 42.4 | | | |
| 3-year | 4.8 | 7.5 | 10.4 | | | |
| 5-year | 0.0 | 0.0 | 0.4 | | | |
| **Race** | | | | | 9.873 | 0.043 |
| White | 261 (84.2) | 659 (89.8) | 1261 (89.0) | 2181 (88.6) | | |
| Black | 20 (6.5) | 32 (4.4) | 80 (5.6) | 132 (5.4) | | |
| Other | 29 (9.4) | 43 (5.9) | 76 (5.4) | 148 (6.0) | | |
| **Sex** | | | | | 19.151 | < 0.001 |
| Female | 131 (42.3) | 270 (36.8) | 434 (30.6) | 835 (33.9) | | |
| Male | 179 (57.7) | 464 (63.2) | 983 (69.4) | 1626 (66.1) | | |
| **Histology** | | | | | 128.552 | < 0.001 |
| SC | 219 (70.6) | 349 (47.5) | 513 (36.2) | 1081 (43.9) | | |
| AD | 91 (29.4) | 384 (52.3) | 903 (63.7) | 1378 (63.7) | | |
| Other | 0 (0.0) | 1 (0.1) | 1 (0.1) | 2 (0.1) | | |
| **Grade** | | | | | 16.506 | 0.002 |
| Well | 30 (9.7) | 36 (4.9) | 80 (5.6) | 146 (5.9) | | |
| Moderately | 116 (37.4) | 286 (39.0) | 624 (44.0) | 1026 (41.7) | | |
| Poorly or Un | 164 (52.9) | 412 (56.1) | 713 (50.3) | 1289 (52.4) | | |
| **Stage** | | | | | 76.644 | < 0.001 |
| Localized | 176 (56.8) | 335 (45.6) | 491 (34.7) | 1002 (40.7) | | |
| Regional | 73 (23.5) | 279 (38.0) | 552 (39.0) | 904 (36.7) | | |
| Distant | 61 (19.7) | 120 (16.3) | 374 (26.4) | 555 (22.6) | | |
| **Surgery** | | | | | 10.382 | 0.006 |
| Surgery | 67 (21.6) | 124 (16.9) | 204 (14.4) | 395 (16.1) | | |
| None | 243 (78.4) | 610 (83.1) | 1213 (85.6) | 2066 (83.9) | | |
| **RT** | | | | | 10.269 | 0.006 |
| RT | 227 (73.2) | 494 (67.3) | 907 (64.0) | 1628 (66.2) | | |
| None | 83 (26.8) | 240 (32.7) | 510 (36.0) | 833 (33.8) | | |

Abbreviation: EC, esophageal cancer; MST, median survival time; RT, radiotherapy.
MST and 1, 3, 5-year survival rate were determined by Kaplan-Meier Analysis.
*MST: log-rank test; others: Chi-square test.
